# Is Nostalgia a Past or Future-Oriented Experience? Affective, Behavioral, Social Cognitive, and Neuroscientific Evidence

**DOI:** 10.3389/fpsyg.2020.01133

**Published:** 2020-06-03

**Authors:** Taylor A. FioRito, Clay Routledge

**Affiliations:** Department of Psychology, North Dakota State University, Fargo, ND, United States

**Keywords:** nostalgia, shared nostalgia, motivation, self-regulation, social cognition

Nostalgia, a sentimental longing for the past, is a common, universal, and highly social emotional experience. Nostalgic reverie is centered around the self, important social connections, and personally meaningful life events (e.g., graduation; Routledge, 2015). In other words, when people bring to mind memories that make them nostalgic, they are revisiting personally meaningful life events shared with loved ones. A growing body of research positions nostalgia as a psychological resource with self-regulatory implications. Negative affective states such as sadness, loneliness, and meaninglessness trigger nostalgia and nostalgia, in turn, enhances well-being, feelings of social connectedness, and perceptions of meaning in life (e.g., Routledge et al., 2013). Building on the behavioral inhibition (BIS/avoidance motivation) and behavioral activation (BAS/approach motivation) regulatory model Carver and White (1994), research also indicates that the activation of avoidance motivation increases nostalgia, which then activates approach motivation (Stephan et al., 2014). In the present analysis, we draw on the current state of the science to propose that nostalgia is ultimately a future-oriented emotional experience. Nostalgia involves reflecting on past experiences but it motivates affective states, behaviors, and goals that improve people's future lives. In the following sections, we briefly review relevant evidence across affective, behavioral, social cognitive, and neuroscientific indicators and close by considering the need for future research focused on nostalgia as a shared experience.

## Future-Oriented Affect

Nostalgia increases general well-being (Routledge et al., 2013) but also positively impacts motivation-relevant affect. For instance, nostalgia increases optimism (Cheung et al., 2013, 2016) inspiration (Stephan et al., 2015) social efficacy (Abeyta et al., 2015) and feelings of purpose in life (Routledge et al., 2011). In addition, as people get older, nostalgia makes them feel youthful and more optimistic about their health (Abeyta and Routledge, 2016). People's written accounts of nostalgic memories also frequently contain themes of appreciation for both the past and hopefulness for the future (Routledge, 2015). In short, nostalgia promotes the types of affective states that mobilize the self for action.

## Future-Oriented Behavior

Critically, nostalgia-induced affective states promote relevant behavior. For instance, health optimism triggered by nostalgia is associated with increased intentions to exercise and eat well, as well as subsequent levels of physical activity (Kersten et al., 2016). Similarly, the social efficacy nostalgia engenders leads to increased social engagement (Abeyta et al., 2015). More broadly, when people experience nostalgia, they are subsequently more likely to engage in prosocial behavior (Stephan et al., 2014), including charitable giving (Zhou et al., 2012). Nostalgia doesn't just make people feel inspired. It drives them to act on their inspiration.

## Future-Oriented Social Cognition

Arguably, the most compelling evidence that nostalgia is a future-oriented emotional experience is its effects on goal-related cognition, since goals are about the future. Nostalgia increases the importance people assign to relationship goals, intentions to pursue the goal of connecting with friends, and the desire to resolve a relationship problem (Abeyta et al., 2015). More broadly, nostalgia increases the motivation to pursue one's most important goal (Sedikides et al., 2017).

Given the social nature of nostalgia, its impact on goals may be strongest in the interpersonal domain. Relatedly, nostalgia's impact on social motivation is moderated by individual differences in attachment-related avoidance (Abeyta et al., 2019). For individuals who rely on relationships for psychological comfort (low attachment-related avoidance), nostalgia increases social goal pursuit. For those who do not rely on relationships for comfort (high attachment-related avoidance), nostalgia decreases social goal pursuit. In sum, nostalgia mobilizes the self, particularly the social self.

## The Motivated Brain

The neuroscience of nostalgia remains limited. Nostalgia proneness is positively related to right-frontal electroencephalogram (EEG) asymmetry, an indicator avoidance motivation and negative emotions (Tullett et al., 2015). Although this evidence is correlational, and thus, we cannot determine causality from it, this finding is in line with past research suggesting that negative emotions and experiences, such as loneliness and meaninglessness, trigger nostalgia as a regulatory resource (e.g., Routledge et al., 2013). More recently, Bocincova et al. (2019) found that nostalgia reduced error related negativity (ERN; a neurological indicator of avoidance motivation) in response to making a mistake in a modified Flanker task, which is consistent with research indicating that nostalgia orients people away from avoidant and toward approach-related psychological states (Stephan et al., 2014). Notably, a preregistered follow-up study did not replicate these findings (FioRito et al., 2020). Further research is required to examine if, and how, nostalgia affects motivation as measured using social neuroscientific paradigms.

## The Need For An Interpersonal Approach: Shared Nostalgia

Although previous research demonstrates that nostalgia is primarily focused on social relationships, almost no work has explored how nostalgia occurs in a social setting. Nostalgia likely frequently implicates social interaction. Indeed, up to 75 percent of conversations may include nostalgic content (Pasupathi et al., 2002; Fivush, 2008; Baron and Bluck, 2009; Beike et al., 2016). Therefore, future research should explore nostalgia as a shared experience. We define shared nostalgia as nostalgia transmitted to at least one other person or exchanged between two or more people. The nature of shared nostalgia needs to be determined. How often does this occur? With whom? What is the role of approach motivation in sharing nostalgic memories with and between others? What social and emotional benefits, if any, can be gained?

We posit that individuals share nostalgia for two purposes: to create and to maintain social connections. The future-oriented qualities of nostalgia may prompt an individual to share a nostalgic memory with an acquaintance to build closeness. Alternatively, those who discuss nostalgic memories with others may “bring online” a social approach motivation, increasing the extent to which the individuals connect. For instance, discussing a nostalgic childhood experience with a new acquaintance could promote self-disclosing behavior in both individuals. Does this boost a desire to deepen the relationship from acquaintances to friends? Moreover, people may discuss a nostalgic memory with others who also experienced in order to maintain the established intimacy. As an example, a couple reflecting together on their first date may feel intimate feelings toward one another. Does this, in turn, increase intentions to stay together?

## Drawing From The Past For The Future

By definition, nostalgia is a past-focused affective experience. A growing body of evidence, however, documents the future-oriented nature of nostalgia. Specifically, people can reference their nostalgic past to remind themselves what it felt to be young (Abeyta and Routledge, 2016) and loved (e.g., Cheung et al., 2013), which, in turn, promotes future-oriented behavior, such as physically caring for oneself (Kersten et al., 2016), connecting with others (e.g., Abeyta et al., 2015), and pursuing goals (e.g., Sedikides et al., 2017). There are deviations from this process, however. For instance, Cheung et al. (2019) recently introduced the concept of anticipated nostalgia. This construct is unique in that it does not rely on the reflections of the past. Instead, anticipated nostalgia is nostalgia for the present and the future (e.g., “I anticipate I will feel nostalgic about my children's childhood in the future”). Critically, Cheung et al. (2019) found that anticipated nostalgia is related to deliberate savoring techniques, such as purchasing souvenirs and documenting moments with pictures. Thus, anticipated nostalgia could be considered a future-focused experience that promotes future-oriented behavior. When discussing the future-oriented nature of nostalgia, individual differences should be considered, as well; not everyone benefits from using nostalgia (e.g., attachment-related avoidance; Wildschut et al., 2010; Juhl et al., 2012; Abeyta et al., 2019). Future research should examine other instances in which nostalgia does not result in future-oriented behavior.

Taken together, when individuals engage in nostalgic reflection, they are not hiding in the past. They are accessing meaningful memories from the past in order to help them approach the future with purpose.

## Author Contributions

TF and CR contributed to the writing of the manuscript.

## Conflict of Interest

The authors declare that the research was conducted in the absence of any commercial or financial relationships that could be construed as a potential conflict of interest.
